# Integrated Transcriptomic and Proteomic Analysis of Primary Human Umbilical Vein Endothelial Cells

**DOI:** 10.1002/pmic.201800315

**Published:** 2019-06-26

**Authors:** Anil K. Madugundu, Chan Hyun Na, Raja Sekhar Nirujogi, Santosh Renuse, Kwang Pyo Kim, Kathleen H. Burns, Christopher Wilks, Ben Langmead, Shannon E. Ellis, Leonardo Collado‐Torres, Marc K. Halushka, Min‐Sik Kim, Akhilesh Pandey

**Affiliations:** ^1^ Center for Molecular Medicine National Institute of Mental Health and Neurosciences Hosur Road Bangalore 560029 Karnataka India; ^2^ Institute of Bioinformatics International Technology Park Bangalore 560066 Karnataka India; ^3^ Manipal Academy of Higher Education Manipal 576104 Karnataka India; ^4^ McKusick‐Nathans Institute of Genetic Medicine Johns Hopkins University School of Medicine Baltimore MD 21205 USA; ^5^ Center for Individualized Medicine and Department of Laboratory Medicine and Pathology Mayo Clinic Rochester MN 55905 USA; ^6^ Neurology Institute for Cell Engineering Johns Hopkins University School of Medicine Baltimore MD 21205 USA; ^7^ Department of Applied Chemistry Kyung Hee University Yongin Gyeonggi 17104 Republic of Korea; ^8^ Departments of Pathology Johns Hopkins University School of Medicine Baltimore MD 21205 USA; ^9^ Sidney Kimmel Comprehensive Cancer Center Johns Hopkins University School of Medicine Baltimore MD 21205 USA; ^10^ High Throughput Biology Center Johns Hopkins University School of Medicine Baltimore MD 21205 USA; ^11^ Department of Computer Science Johns Hopkins University Baltimore MD 21218 USA; ^12^ Center for Computational Biology Johns Hopkins University Baltimore MD 21205 USA; ^13^ Department of Biostatistics Johns Hopkins Bloomberg School of Public Health Baltimore MD 21205 USA; ^14^ Lieber Institute for Brain Development Johns Hopkins Medical Campus Baltimore MD 21205 USA; ^15^ Department of New Biology DGIST Daegu 42988 Republic of Korea; ^16^ Department of Biological Chemistry Johns Hopkins University School of Medicine Baltimore MD 21205 USA; ^17^ Department of Oncology Johns Hopkins University School of Medicine Baltimore MD 21205 USA

**Keywords:** allelic expression, coding SNP, mass‐spectrometry, RNA‐seq, proteoform, proteogenomics, splice variants, transcriptome

## Abstract

Understanding the molecular profile of every human cell type is essential for understanding its role in normal physiology and disease. Technological advancements in DNA sequencing, mass spectrometry, and computational methods allow us to carry out multiomics analyses although such approaches are not routine yet. Human umbilical vein endothelial cells (HUVECs) are a widely used model system to study pathological and physiological processes associated with the cardiovascular system. In this study, next‐generation sequencing and high‐resolution mass spectrometry to profile the transcriptome and proteome of primary HUVECs is employed. Analysis of 145 million paired‐end reads from next‐generation sequencing confirmed expression of 12 186 protein‐coding genes (FPKM ≥0.1), 439 novel long non‐coding RNAs, and revealed 6089 novel isoforms that were not annotated in GENCODE. Proteomics analysis identifies 6477 proteins including confirmation of *N*‐termini for 1091 proteins, isoforms for 149 proteins, and 1034 phosphosites. A database search to specifically identify other post‐translational modifications provide evidence for a number of modification sites on 117 proteins which include ubiquitylation, lysine acetylation, and mono‐, di‐ and tri‐methylation events. Evidence for 11 “missing proteins,” which are proteins for which there was insufficient or no protein level evidence, is provided. Peptides supporting missing protein and novel events are validated by comparison of MS/MS fragmentation patterns with synthetic peptides. Finally, 245 variant peptides derived from 207 expressed proteins in addition to alternate translational start sites for seven proteins and evidence for novel proteoforms for five proteins resulting from alternative splicing are identified. Overall, it is believed that the integrated approach employed in this study is widely applicable to study any primary cell type for deeper molecular characterization.

## 1 Introduction

The vascular endothelium is an important organ system to maintain vascular homeostasis in intravascular and extravascular spaces by modulating blood vessel tone.^1^ Endothelial cells produce essential autocrine and endocrine molecules essential for regulating local cellular growth and deposition of extracellular matrix.^2^ Changes to the endothelium contribute to pathogenesis of diseases such as coronary artery disease, atherosclerosis, and other vascular diseases.^3^, ^4^, ^5^ Endothelial dysfunction leads to hypertension, hypercholesterolemia, diabetes, septic shock, insulin resistance, chronic kidney failure, and Behcet's disease.^4^, ^6^, ^7^, ^8^ Recent reports also demonstrate emerging role of endothelial cells in cancer invasion and spontaneous metastasis.^9^, ^10^ For these reasons, the study of human endothelium is important to elucidate their molecular profile underlying the key role in vascular development and maintenance.

The human umbilical vein endothelial cells (HUVEC) are an excellent system to study vascular diseases and the effect of drugs using in vitro cell culture approaches.^11^, ^12^ Early proteomics analysis of HUVECs were carried out by 2D gel electrophoresis and LC‐MS/MS methods which identified a few hundred proteins.^11^, ^13^, ^14^, ^15^ Large‐scale analysis of HUVECs has been enabled by technological developments in mass spectrometry which led to the identification of thousands of proteins under various perturbations.^9^, ^16^, ^17^, ^18^, ^19^ The role of increased expression of cell adhesion molecules on HUVECs is well documented under the conditions of atherosclerosis and other pathological conditions.^20^, ^21^, ^22^ Deep characterization by transcriptomics and proteomics analysis of a few human cell types has been attempted although the large majority of them are cancer cell lines.^23^, ^24^, ^25^, ^26^ Further, although many microarray‐based gene expression studies and some RNA‐seq studies have been conducted on HUVECs, deep characterization of HUVEC transcripts and its integration with proteomics data has not yet been published.

Recent advances in high‐throughput methods of global gene expression profiling allow systematic study of flow of genetic information encoded by the genome into transcripts and proteins across human tissues.^27^, ^28^, ^29^ Mammalian cell culture systems enable studying the physiology and pathology of human diseases in the laboratory. A systematic study of vascular diseases was initially limited by the non‐availability of human endothelial tissue until a culture‐based method was first developed.^12^ Our laboratory has previously described benefits of such approach by molecular profiling of tissues by integrated analysis of transcriptomics and proteomics in cells, tissues, and organisms.^30^, ^31^, ^32^, ^33^ In this study, we have undertaken expression profiling of endothelial cells from primary cultures of HUVECs. A systematic analysis of gene expression analysis in the context of other human tissues and integrated analysis of mRNA and protein level information was carried out. We started by profiling the expressed genes at the mRNA level and undertook deep analysis of proteomics data to identify protein cleavage sites and multiple post‐translational modifications (PTMs). In addition, we specifically sought to identify coding single nucleotide polymorphism (SNPs), novel protein N‐termini and alternate exon–exon splice junctions by this integrated analysis. Overall, we demonstrate the capability of integrated analysis of RNA‐seq and proteomics data to better characterize the molecular profile of endothelial cells.

**Significance Statement**

Primary cell cultures are an excellent system to study biological systems under controlled conditions. An understanding of genes and proteins that are expressed in any cell type is of utmost importance in studying molecular alterations in disease states and in response to various stimuli. We undertook deep sequencing of mRNAs and carried out a comparison against other human tissues to identify cell‐type‐specific genes as well as novel long non‐coding RNAs. A comparison of the transcripts against human gene expression data for 53 tissues catalogued by the Genotype‐Tissue Expression (GTEx) project revealed a number of HUVEC‐specific genes. Proteomic profiling by high‐resolution mass spectrometry led to identification of nearly 6500 proteins and many post‐translational modifications and novel protein species using a comprehensive integrated approach. By creating a custom database of proteins containing sample‐specific single amino acid variants (SAAV), we also identified alternate protein alleles for more than 200 protein‐coding genes. This catalog of expressed genes and proteins in HUVECs will serve as a useful reference for future studies.

## 2 Experimental Section

### 2.1 Cell Culture

Primary HUVECs were obtained from Lonza (Catalog # CC‐2517) and cultured as per the manufacturer's specifications. Briefly, 5 × 10^5^ cells were seeded into a 10‐cm cell culture dish in endothelial basal medium (EGM BulletKit, Catalog # CC‐3124, Lonza) supplemented with growth factors including bovine brain extract, (BBE, w/ heparin), hEGF, hydrocortisone, ascorbic acid, GA‐1000 (gentamicin, amphotericin B), and 2% FBS. The cells were grown in 5% CO_2_ at 37 °C for six passages and harvested for protein and RNA extraction.

### 2.2 RNA Isolation and Sequencing

Total RNA was isolated from HUVECs using RNeasy mini kit (Qiagen) according to the manufacturer's instructions. Total RNA isolated was quantified and assessed for quality using Agilent BioAnalyzer. High‐quality DNase treated total RNA from HUVECs was used for library preparation using TruSeq stranded mRNA library preparation kit from Illumina and sequenced on HiSeq2500 according to the manufacturer's guidelines. Briefly, mRNA enrichment from total RNA was carried out by polyA tail capture using oligo‐DT beads prior to heat‐based fragmentation and elution of mRNA. The eluted fragments were converted to cDNA and their 3' ends were adenylated. Adapters consisting of index sequences were then ligated to the adenylated cDNA, prior to limited cycle PCR amplification of the adapter ligated cDNA fragments. Amplified libraries were size‐selected, clustered, and sequenced after analyzing the quality of the library using BioAnalyzer. Quality checked library was sequenced using Illumina HiSeq 2500 in the high‐output mode to obtain 100 bp paired‐end reads.

### 2.3 Transcriptome Data Analysis

**Figure 1:**
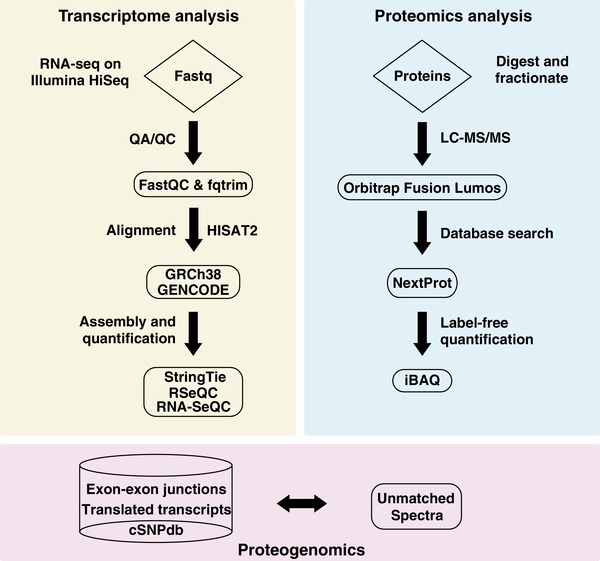
Schematic of data analysis workflow employed in this study. Top left and right panels describe the analysis steps and tools employed in transcriptomic and proteomic analysis, respectively. Bottom panel describes the integrated analysis of proteomics and proteogenomics carried out in this manuscript.

Raw data generated from Illumina was demultiplexed and base calling was made using bcl2fastq v1.8.4 of CASAVA v1.8 (Illumina). Quality assessment of raw fastq data was then carried out using FastQC v0.11.5 to detect systemic artifacts in sequencing. Raw read pairs were subjected to trimming using Fqtrim v0.9.5^34^ by clipping low‐quality bases (Q <20 and length <35 bp) from ends. The human genome version GRCh38 (hg38) and gene annotations from GENCODE release 26^35^ were used as reference. Quality processed data was then aligned against the GRCh38 genome index using HISAT2 v2.0.1 aligner.^36^ Alignment was carried out in default “–end‐to‐end” mode with sensitive options (‐D 15 ‐R 2 ‐N 0 ‐L 22 ‐i S,1,1.15). Alignment data from HISAT2 was converted, sorted, and indexed using samtools v1.3.1.^37^ Transcript assembly and quantification were carried out using StringTie v1.3.3 package.^38^ StringTie was run with default parameters and GENCODE release 26 annotation GTF was supplied to guide assembly of transcripts. A detailed workflow of transcriptome analysis is shown in Figure 1. We also followed GTEx pipeline to facilitate fair comparison with their tissue expression data.^27^ In summary, quality processed data was similarly aligned against the human genome version GRCh37 (hg19) supplemented with GENCODE release 19 annotations. Gene level expression measured as reads per kilobase of transcript per million mapped reads (RPKM) values were obtained using RNA‐SeQC v1.1.8.^39^ Median RPKM data across the GTEx (release v6p) tissues for all human genes was downloaded for comparison.^27^

### 2.4 Protein Extraction, Digestion, and Labeling

The HUVEC cells were harvested by scraping the cells in lysis buffer containing 4% sodium dodecyl sulfate in 100 mm Tris, pH 7.6 after washing with cold phosphate buffered saline thrice. Subsequently, the cells were sonicated with a probe sonicator for 20 s followed by centrifuging at 16 000 × *g* for 5 min at room temperature. The protein concentration of the cleared lysate was estimated using a bicinchoninic acid assay. Two hundred micrograms of proteins were reduced and alkylated with 2.5 mm tris(2‐carboxyethyl) phosphine, 5 mm chloroacetamide, and 100 mm triethylammonium bicarbonate (TEAB) at room temperature for 30 min with shaking at 800 rpm. After the reduction and alkylation, the proteins were precipitated by adding chilled acetone (−20 °C) followed by incubating at −20 °C for 2 h. The sample was centrifuged at 17 000 × *g* at 4 °C for 5 min, washed with methanol twice and dried in the vacuum. For enzyme digestion of the proteins, 10 µg of sequencing grade trypsin in 0.5 mL of TEAB was added to the dried protein followed by shaking at 4 °C at 1000 rpm for 1 h. The enzyme digestion was conducted at 37 °C overnight. The peptides were fractionated by StageTip strong cation exchange chromatography (SCX) as described previously.^40^ Briefly, the peptides were reconstituted in 200 µL of 1% trifluoroacetic acid (TFA) and centrifuged at 16 000 × *g* for 5 min. Two plugs of SCX disk (Polystyrene‐divinylbenzene copolymer modified with sulfonic acid) was used for a preparation StageTip column. The StageTip column was activated by loading 100 µL of acetonitrile (ACN) by centrifuging the StageTip column at 2000 × *g* until all the ACN pass through the column. Eighty micrograms of peptides were loaded on the StageTip column and centrifuged at 2000 × *g* until all the sample solution passed through the column. For the elution of the first fraction, 80 µL of SCXx1 (50 mm ammonium acetate (AA), 20% v/v ACN, 0.5% v/v formic acid (FA)) was loaded and centrifuged at 2000 × *g* until all the solution passed through the column. This elution was repeated for SCXx2 (75 mm AA, 20% v/v ACN, 0.5% v/v FA), SCXx3 (125 mm AA, 20% v/v ACN, 0.5% v/v FA), SCXx4 (200 mm AA, 20% v/v ACN, 0.5% v/v FA), SCXx5 (300 mm AA, 20% v/v ACN, 0.5% v/v FA), and Buffer X (5% v/v ammonium hydroxide, 80% v/v ACN). The eluted samples were dried under vacuum.

### 2.5 Mass Spectrometry Analysis

The dried peptides were reconstituted in 0.5% FA and analyzed on an Orbitrap Fusion Lumos Tribrid Mass Spectrometer coupled with the EASY‐nLC 1200 nano‐flow liquid chromatography system (Thermo Fisher Scientific). The peptide fractions were loaded on an Acclaim PepMap100 Nano‐Trap Column (100 µm  ×  2 cm, Thermo Fisher Scientific) packed with 5 µm C_18_ particles at a flow rate of 5 µl min^−1^. Peptides were resolved at 250 nL min^−1^ flow rate using a linear gradient of 10–35% solvent B (0.1% FA in 95% ACN) over 150 min on an EASY‐Spray column (50 cm × 75 µm ID), PepMap RSLC C18, and 2 µm C_18_ particles (Thermo Fisher Scientific), which was fitted with an EASY‐Spray ion source that was operated at a voltage of 2.2 kV. Mass spectrometry analysis was carried out in a data‐dependent manner with a full scan in the mass‐to‐charge ratio (*m*/*z*) range of 300–1500 in the “Top Speed” setting, 3 s per cycle. MS1 scans were measured at a resolution of 120 000 at an *m*/*z* of 200. MS/MS scans were acquired by fragmenting precursor ions using the higher‐energy collisional dissociation method and detected at a mass resolution of 30 000, at an *m*/*z* of 200. Automatic gain control for MS1 was set to one million ions and for MS/MS was set to 0.05 million ions. A maximum ion injection time was set to 100 ms for MS1 and MS/MS. Higher‐energy collisional dissociation was set to 32 for MS/MS. The precursor isolation window was set to 1.6 Da with the offset of 0.5. Dynamic exclusion was set to 30 s, and singly charged ions were rejected. Internal calibration was carried out using the lock mass option (*m*/*z* 445.1200025) from ambient air.

### 2.6 Proteomics Data Analysis

The raw files were analyzed using Proteome Discoverer 2.1 (Thermo Scientific, Bremen, Germany) with SEQUEST and Mascot (v2.2.06) search engines. The mass spectrometry data was searched against neXtProt (release date: January 17, 2018) human protein database^41^ and common contaminants. Trypsin was specified as protease allowing up to two missed cleavages. Carbamidomethylation of cysteine as fixed modification and acetylation of protein N‐termini, oxidation of methionine, and phosphorylation of serine/threonine/tyrosine amino acids as variable modifications were specified. The precursor mass tolerance was set to 10 ppm and fragment ion tolerance was limited to 0.02 Da. Initial peptide spectral matches are further validated by Percolator to ascertain the assignments using estimated posterior‐error probabilities (PEP). Only tryptic peptides longer than seven amino acids with up to two missed cleavages were considered. The data were searched in target‐decoy approach and filtered by applying 1% false discovery rate (FDR) at peptide and protein levels. Unmatched spectra from first iteration of database search against protein reference database were exported as Mascot genetic format (MGF) file for downstream analyses. Additional analysis for identification of PTMs in unmatched spectra was made by carrying out separate searches by specifying variable modifications of lysine acetylation; lysine and arginine methylation, dimethylation, and trimethylation; and lysine ubiquitylation while keeping other parameters the same as in the initial search.

### 2.7 Integrated Analysis

All genomics analyses were carried out using GRCh38 (hg38) as reference genome unless specified. Exon–exon splice junctions from the alignment data were detected and annotated using “junction_annotation.py” tool of RSeQC v2.6.4.^42^ All novel category of splice junctions were queried using Snaptron^43^ to detect their occurrence and coverage details in all samples across the recount2^44^ resource. recount2 summarizes splice‐junction evidence from 50 099 human run accessions in the Sequence Read Archive (SRA), 9662 accessions from 551 individuals in the GTEx project and 11 350 accessions from 10 340 individuals in the cancer genome atlas (TCGA) project. For each accession and each splice junction, recount2 records the number of split‐read alignments supporting the junction in the accession. Because the analysis pipeline used to obtain these counts does not use a gene annotation, neither annotated nor unannotated junctions are unduly favored. Snaptron was used to rapidly query the recount2 resource to obtain a junction's evidence profile across all accessions. This profile can be further summarized into measures of the junction's prevalence and tissue specificity.

Novel splice junctions identified were further processed to extract 90 bp sequences from the exon ends to produce tryptic peptides spanning exon–exon junction that correspond to lengths generally detectable by mass spectrometry. Nucleotide sequences derived from exon–exon junctions were translated into a peptide database. Sample‐specific genetic variation was detected from aligned RNA‐seq data using pipeline described previously.^45^ In brief, duplicated reads in aligned BAM files were filtered using Picard v2.10.9, realigned around known indels and variants called using GATK v3.6‐0. Variants identified were annotated using Ensembl VEP^46^ against Ensembl reference annotations (release 90) and filtered for non‐synonymous variations in expressed genes. A custom database of protein sequences with single amino acid polymorphisms (SAAV) was then constructed by incorporating the non‐synonymous amino acid changes. Similarly, all assembled transcripts from StringTie analysis were translated in three frames to generate a putative ORF database. Unmatched tandem mass spectral data exported in the previous step was searched separately against all these custom databases created from RNA‐seq analyses.

### 2.8 Validation of Novel Findings by Synthetic Peptides

Peptide sequences that corresponded to missing proteins or novel observations were generated as synthetic peptides (JPT Peptide Technologies, Berlin, Germany). The synthetic peptides were reconstituted with 50% ACN and 0.1% FA. Approximately 1 pmol of each peptide was mixed in an equimolar ratio and analyzed on an Orbitrap Fusion Lumos Tribrid mass spectrometer (Thermo Fisher) as described above. The experimentally obtained fragmentation spectra for novel peptide identifications were validated by aligning them with those obtained from the synthetic peptides using in‐house software written in the Python programming language.

### 2.9 Data Availability

Transcriptome data including both raw fastq files and assembled transcript annotations were deposited in NCBI's Gene Expression Omnibus (GEO) and is accessible through GEO Series accession number GSE115536 (http://www.ncbi.nlm.nih.gov/geo/query/acc.cgi?acc=GSE115536). And raw mass spectrometry (MS) files used in this experiment have been uploaded to the ProteomeXchange Consortium (http://proteomecentral.proteomexchange.org) via the PRIDE partner repository with the dataset identifier PXD009687.

## 3 Results and Discussion

### 3.1 RNA‐Seq‐Based Gene Expression Analysis of HUVEC Transcriptome

**Figure 2:**
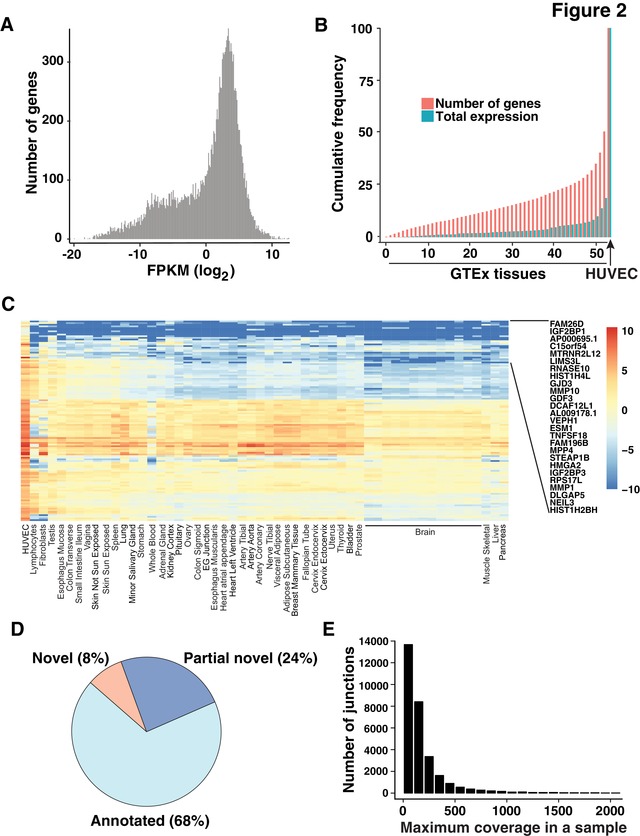
Summary of results from transcriptomic analysis. A) Distribution of FPKM (log_2_) over the protein‐coding genes. B) Histogram shows the relative expression of genes expressed across the number of human tissues in GTEx. C) Heatmap showing the relative expression of cell type enriched protein‐coding genes across the human tissues in GTEx. D) Distribution of identified splice junctions. Novel splice junctions were formed by novel splice donor and acceptor sites whereas partial novel junctions were form by a novel donor or acceptor site. E) Comparison to coverage of splice junctions in over 70 000 human RNA‐seq accessions collected in recount2.

Sequencing of the poly(A) library on Illumina platform resulted in the generation of 145 million paired‐end reads of 101 bp length. Sequenced reads were of high quality (Q ≥20) and consistent over the entire tiles of sequencing lane. No significant contamination by known sequencing adapters in the sequenced data was detected. Quality processing of fastq data eliminated about 8 million poor quality read pairs (Phred, Q <20 and length <35 bp). Alignment of processed data against human genome reference (GRCh38) using HISAT2 resulted in mapping 96.5% input reads. Over 80% of aligned read pairs could be mapped unambiguously to the annotated gene regions and were used for quantifying the gene expression. Among the read‐pairs mapping uniquely to the genome, 98.6% were within the regions annotated as protein‐coding genes. This also validates the efficient enrichment of mRNA molecules by poly(A) selection method of library preparation. The distribution of fragments per kilobase of transcript per million mapped reads (FPKM) over the annotated protein‐coding genes follows a bimodal distribution as shown in Figure 2A. Out of 19 846 annotated protein‐coding genes, a total of 12 186 were detectable (FPKM ≥0.1)^47^ while the remainder of the genes were considered to be very low in expression or not expressed (FPKM <0.1).

In the transcriptome analysis, non‐coding RNA (ncRNA) genes were the largest group after protein‐coding genes. Long intergenic non‐coding RNAs (lincRNAs) are the major subclass of ncRNAs which are transcribed from non‐coding regions between protein‐coding genes and are usually longer than 200 bp.^48^ Analysis of transcriptomic data has revealed expression of several thousand lincRNAs in many human tissues.^49^ A pipeline previously described by our group identified ≈8000 lincRNAs expressed in primary human monocytes including 1000 novel lincRNAs.^50^ Following the same pipeline for this study, analysis of HUVECs RNA‐seq data identified 1029 known and 439 novel lincRNAs with FPKM ≥0.1. Expression levels of several known lincRNAs correlate with previous studies showing abundant levels of MALAT1, TUG1, MEG3, LINC00657, and LINC00493 in HUVECs.^51^ Expression of MALAT1 in endothelial cells has previously been shown to be essential for proliferation and vascularization.^51^

Global identification and quantification of gene expression levels across tissues allow studying the tissue‐/cell‐type‐specific functions. The GTEx portal provides expression levels of genes in 53 human tissues from multiple individuals. To identify endothelial‐cell‐specific gene expression, we compared our RNA‐seq data with the transcriptomic data from GTEx (v6p). We followed the pipeline used in GTEx RNA‐seq data analysis to align and quantify gene expression.^27^ The histogram in Figure 2B shows the cumulative frequency of genes that are expressed in HUVECs among the 53 tissue types represented in GTEx. Half of the genes expressed in the endothelial cells, which are ubiquitously expressed in all other human tissues contribute to >80% of the total mRNA, which is consistent with previous studies.^47^, ^52^, ^53^ Hierarchical clustering of log_2_ transformed RPKM values of annotated protein‐coding genes was then carried out to group genes and tissues with similar gene expression. Gene expression profile of HUVEC was found to be somewhat similar to lymphocytes, whole blood, heart followed by muscular tissue types. To identify HUVEC‐specific gene expression against the tissues studied in GTEx, we employed a method described previously.^54^ Based on this, only 48 genes were found highly enriched in HUVECs. A heatmap showing the relative expression of HUVEC enriched genes is shown in Figure 2C and listed in Supporting Information S1. Pathway analysis revealed proteins significantly overrepresented in the angiogenesis process (*p*‐value = 9.3 × 10^−5^), which include ANGPT2, CALCRL, ESM1, ECSCR, PGF, and TIE1 that are highly enriched. This allowed us to identify the likely cell–type‐specific roles associated with endothelial cells and to understand their relevance in development and functioning of vascular systems.

Human cells orchestrate complex cellular regulation through expression of diverse transcripts from ≈20 000 genes. Thus, we investigated the diversity of the transcripts expressed in HUVECs. Transcript reconstruction and quantification from aligned RNA‐seq data was carried out using StringTie guided by the GENCODE reference annotations. A total of 35 068 transcripts identified from 12 011 genes were annotated in GENCODE with a mean of three transcripts expressed per gene. Additionally, 6089 new alternate isoforms were detected in HUVECs. Similar analysis focused at exon–exon junctions using RSeQC^42^ identified many novel splice junctions not annotated in GENCODE. Of the 30 362 alternate splice junctions identified, 24% were partially novel resulting from alternate splice acceptor or donor sites while 8% were completely novel as shown in Figure 2D. All novel splice junctions were queried using Snaptron.^43^ Specifically, novel junctions were used to query Snaptron's SRAv2, GTEx, and TCGA compilations, which together index over 70 000 human RNA‐seq runs.^44^ Figure 2E shows the distribution of maximum read count in any sample supporting the 30 362 query junctions identified from HUVECs. Most of the junctions identified in HUVECs were also observed in at least one RNA‐seq run that are publicly available.

### 3.2 Annotation of HUVEC Proteins Through Mass Spectrometry‐Derived Proteomic Data

**Figure 3:**
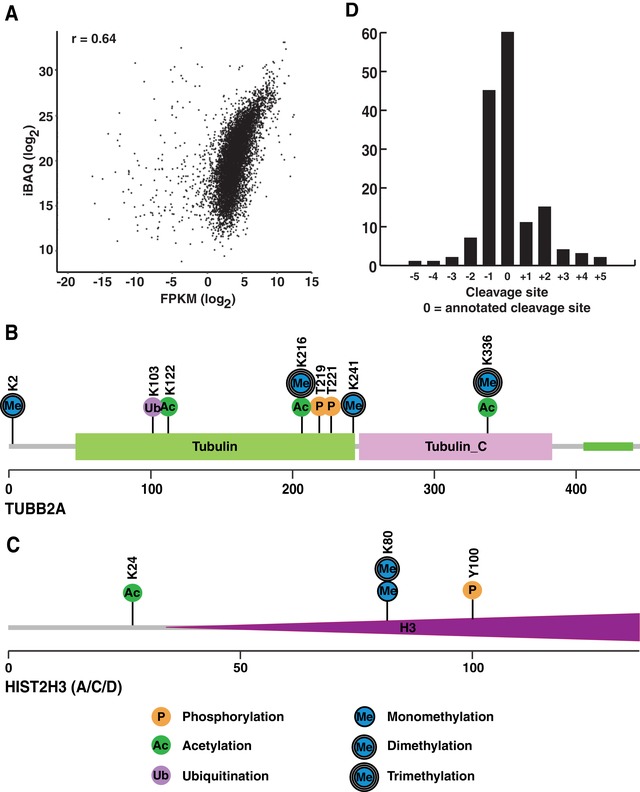
Integrated analysis of proteomics data. A) Scatter plot showing the comparison of gene expression at mRNA and protein levels. Schematic representation of B) TUBB2A and C) HIST2H3 proteins with identified PTMs. D) Histogram of number of proteins identified with annotated and alternate signal peptide cleavage sites. Alternate cleavage sites flanking to annotated (0) cleavage site.

Advancements in mass‐spectrometry‐based methods now allow identification of thousands of proteins in biological specimens. To catalog proteins expressed in HUVECs, we carried out high‐resolution LC‐MS/MS analysis followed by bioinformatics analysis to identify, quantify, and compare against expression at mRNA level. Strong cation‐exchange‐based peptide fractions of HUVEC whole cell lysate protein digests were analyzed in duplicate on a Thermo Scientific Orbitrap Lumos instrument. The LC‐MS/MS analysis generated over one million spectra. Using the target‐decoy strategy for database searching by SEQUEST and Mascot search algorithms, we identified 463 451 peptide spectrum matches (PSMs) at a FDR of 1%. Applying the parsimony principle, a total of 75 903 detected peptides were grouped into 6477 unique human proteins (6641 isoforms) at 1% protein FDR (Supporting Information S2). The intensity‐based absolute quantification (iBAQ) approach^55^ was used to estimate the abundance of proteins identified in this study. Briefly the normalized intensities of PSMs from each MS analysis were summed for peptides which were grouped into proteins. The iBAQ measures of proteins were then compared against the FPKM values of genes identified from the transcriptome analysis. Similar to previous reports, the correlation between the mRNA and protein expression was low with Pearson correlation coefficient, *r* = 0.64 and a scatter plot between the log_2_ transformed FPKM and iBAQ is shown in the Figure 3A. Out of a total 150 756 annotated splice junctions in GENCODE, we identified 14 384 exon–exon junction spanning peptides for 4970 proteins. Over 1000 peptides uniquely represented 310 high confident protein isoforms, also referred to as proteoforms. The following sections describe the role of proteins involved in endothelial cell function, PTMs, and proteolytic cleavage.

One class of functional molecules that are of interest in the vascular endothelium is adhesion molecules. Vascular endothelial cell adhesion molecules play key roles in inflammation and immune responses by interacting with the circulating leukocytes. Several types of adhesion molecules including integrins, selectins, and immunoglobulin superfamily members are involved in this process. For example, endothelial cells show expression of selectins and immunoglobulin superfamily members upon activation by integrins on circulating lymphocytes. Expression of many other adhesion molecules is similarly tightly regulated by proinflammatory cytokines and other molecules that are released by lymphocytes.^56^ We investigated the kind of cell adhesion molecules that were expressed in HUVEC. We observed PECAM1 (CD31), a marker of endothelial cells and platelets, to be highly expressed at both mRNA and protein levels. Among members of the immunoglobulin superfamily, mRNA and protein expression levels were high for ICAM‐2 followed by ICAM‐1, ICAM‐3, and VCAM‐1, as described previously.^6^ Only one member of selectins, E‐selectin was identified at the transcript and protein levels, which is again consistent with previous observations that other selectins: P‐selectin and L‐selectin were not identified in HUVECs.^21^ A complete list of endothelial cell adhesion molecules identified in this study along with their expression levels is presented in Table 1.

**Table 1:** Selected cell adhesion molecules identified in HUVECs

Gene symbol	Protein	PSMs	FPKM
*ICAM2*	Intercellular adhesion molecule 2	82	278.91
*PECAM1*	Platelet endothelial cell adhesion molecule	431	244.76
*ESAM*	Endothelial cell selective adhesion molecule	61	131.91
*JAM3*	Junctional adhesion molecule C	14	35.27
*F11R*	Junctional adhesion molecule A	54	30.70
*NRCAM*	Neuronal cell adhesion molecule	35	11.61
*BCAM*	Basal cell adhesion molecule isoform	13	7.65
*ICAM1*	Intercellular adhesion molecule 1	59	5.91
*CEACAM19*	Carcinoembryonic antigen‐related cell adhesion molecule 19	–	3.07
*CADM4*	Cell adhesion molecule 4	–	2.28
*SELE*	E‐selectin	3	1.86
*ICAM3*	Intercellular adhesion molecule 3	–	1.51
*CEACAM21*	Carcinoembryonic antigen‐related cell adhesion molecule 21	–	1.06
*CDON*	Cell adhesion molecule‐related/downregulated by oncogenes	–	0.95
*CADM3*	Cell adhesion molecule 3	7	0.48
*CEACAM1*	Carcinoembryonic antigen‐related cell adhesion molecule 1	–	0.45
*EPCAM*	Epithelial cell adhesion molecule	–	0.33
*JAM2*	Junctional adhesion molecule B	–	0.27
*VCAM1*	Vascular cell adhesion molecule 1	–	0.21
*CADM1*	Cell adhesion molecule 1	–	0.18

### 3.3 Deep Analysis of HUVEC Proteome for Missing Proteins and Post‐Translational Changes

Curated databases such as UniProt, RefSeq, HPRD, neXtProt^28^, ^41^, ^57^, ^58^ and many other similar resources have been designed to characterize human proteins in detail and provide supporting evidence that has been accumulated in the published literature. The Human Proteome Project (HPP) organized by the Human Proteome Organization (HUPO) recommends using neXtProt as primary knowledgebase for the analysis of proteomics data and has laid down guidelines to classify proteins at five levels of protein evidence (PE 1–5). The latest HPP guidelines 2.1.0 require identification of two uniquely mapping non‐nested peptides with a minimum length of nine amino acids to qualify a protein as known (PE1). Exceptions are allowed only for the proteins that can never meet this requirements.^59^, ^60^ All proteins annotated with PE 2–4 level of evidence are classified as “missing proteins.” To accumulate evidence at the protein level for missing proteins, we compared the proteins identified in this study against list of “missing proteins” in neXtProt (release date: January 17, 2018). At least two unique peptides were identified for 11 “missing proteins” while 22 “missing proteins” were identified with only one unique peptide. Peptide evidence for five missing proteins which include HSP90AA4P, NACAP1, MAP1LC3B2, SNRPGP15, and CBWD6 were further validated by synthetic peptides. We believe deep proteomic profiling of unsampled tissues and cell types are more likely to contribute to the knowledge of “missing proteins” as demonstrated here.

PTMs represent another mechanism that diversifies the proteoforms produced from the limited number of human genes.^61^ Proteomic analysis of cellular proteomes without any enrichment step, as in this study, permits detection of a variety of PTMs in an unbiased fashion. To catalog PTMs in the HUVEC proteome, we searched unmatched tandem mass spectra from first‐pass search against the neXtProt protein database to minimize enormous search space, compute time, and false peptide identifications. By specifying variable PTMs of amino acids, we identified thousands of peptides containing at least one PTM. This included 46 ubiquitination sites, 91 lysine acetylation sites, and 231 mono‐/di‐/tri‐methylation sites on lysine or arginine residues in addition to 1034 phosphosites identified previously from first‐pass search. Overall, 762 post‐translationally modified proteins were identified in this study (Supporting Information S3). This approach shows the possibility of reliable identification of multiple PTMs from protein samples without employing any enrichment method or depletion strategies. Searching for multiple PTMs in a routine workflow is not feasible as it increases the search space and thereby decreases the sensitivity of peptide and protein identifications. Exporting the unmatched mass spectra from routine search workflow overcomes this limitation by making the PTM search feasible and for carrying out proteogenomics analyses against custom databases. A schematic of protein domains along with multiple PTMs identified is shown for tubulin beta‐2A (Figure 3B) and histone H3.2 (Figure 3C) proteins. Other proteins with multiple PTMs include ACTB, ACTG1, MAP1B, AHNAK, VIM, NES, SRRM2, ACTA1, and AKAP12. Most proteomics data analysis pipelines do not search for multiple PTMs owing to the widened search space and time. Using the iterative approach described in this study, we could identify several common PTMs from a routine sample that was not processed for enriching any PTMs. We believe proteomics groups will benefit from this approach and make it as a practice in routine proteomics analysis. Following this approach might also benefit the research community by sharing and depositing various PTMs in public repositories.

**Figure 4:**
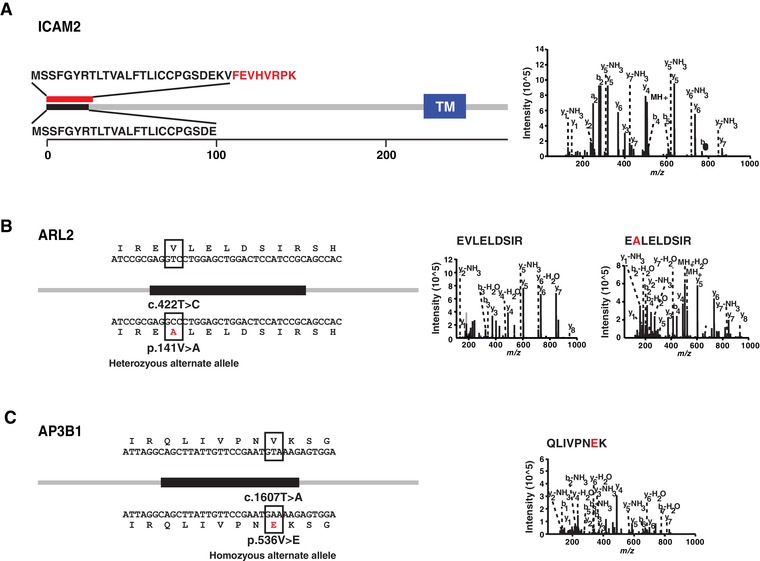
A) A schematic showing the ICAM2 protein with annotated (bottom) and alternate (top) signal peptide cleavage sites. Schematic of proteins with alternate allele(s) identified from cSNP database search at mRNA and protein levels: Both wild type and alternate alleles were found for B) ARL2 protein while only homozygous alternate allele was found for C) AP3B1 protein.

The localization of proteins to membranes is determined in large part by the presence of signal peptides at the protein N‐termini. Manually curated as well as predicted signal peptides and corresponding cleavage sites are available for proteins from HPRD,^62^ SMART,^63^ and SignalP^64^ resources. Our group has previously identified a number of signal peptides along with their cleavage sites from analysis of hemodialysis and plasma samples.^65^, ^66^ To identify such events, we permitted semi‐tryptic specificity in the search against a database of curated N‐terminal signal peptides. We identified 122 proteins processed N‐termini as the signal peptides were already cleaved in the mature proteins (Supporting Information S4). Of these, annotated cleavage sites were observed in 60 proteins while the cleavage sites were novel (cleavage within 5 amino acids of the annotated cleavage site) for 62 proteins. Figure 3D shows a histogram of the number of novel cleavage sites. Interestingly, 13 proteins were identified with both annotated and alternate cleavage sites. These proteins include: CD59, CD93, F11R, ICAM2, MCAM, P4HB, PDIA3, PRDX4, RPN2, SERPINE1, THBS1, TM9SF4, TMED10, and TMED7. As expected, the majority of these proteins are associated with the membrane. As an illustration, a schematic of ICAM2, which was identified with two signal peptide cleavage sites is shown in Figure 4A. Though the number of proteins identified with cleavage sites was small, routine analysis might reveal novel mechanisms of protein trafficking and cellular sorting.

### 3.4 Integrated Analysis of Transcriptomic and Proteomic Data Reveals Novel Protein Variants

Next‐generation sequencing of RNA from the same cells for which the proteomic analysis was carried out offers the ability to capture sample‐specific genetic variants that were missed when a reference protein database is used for searching. Nucleotide‐level resolution of RNA‐seq methods also captures sample‐specific sequence variation in transcripts. We investigated the extent of coding SNPs in the proteomics data by cataloging the variants in the HUVEC sample against the human genome reference (GRCh38). This resulted in the identification of 6279 non‐synonymous variants in 3933 protein‐coding genes. Proteomics data analysis by searching against the custom protein database containing SAAVs encoded in the transcriptome resulted in identification of 245 peptides containing an amino acid variant derived from 207 proteins (Supporting Information S5). For 71 of these 207 proteins, a peptide encoded by the corresponding reference allele was also identified indicating the heterozygous status of allelic expression at the protein level. For example, in the case of ARL2, we identified both reference and cSNP peptides (E[V/A]LELDSIR) as shown in Figure 4B. For 85 proteins, only the alternate allelic form was detected at the protein level where the mRNAs also showed homozygous status of the variant allele. In the case of AP3B1 shown in Figure 4C, as expected we identified only the cSNP peptide (QLIVPNEK) but not the reference peptide. For 51 proteins, we detected only the cSNP encoded peptide although transcripts from both alleles were expressed. These observations demonstrate the existence of allele‐specific expression at the protein level and the need for an integrated approach of transcriptomics and proteomics analysis to identify them.

The majority of protein translation sites in the reference databases are derived from conceptual translation of putative open reading frames. A growing number of studies indicate that cells express diverse alternative translation start sites.^28^, ^67^, ^68^, ^69^, ^70^ In addition, there exist cell‐/tissue‐specific translation initiation sites (TISs).^67^ About 90% of human proteins have N‐terminal acetylation, which can serve as a signature for a TISs. To identify both acetylated and non‐acetylated N‐termini of proteins, we carried out database searches for protein N‐termini with and without acetylation as a variable N‐terminal modification. In all, we identified 1118 N‐terminally acetylated peptides mapping, thereby validating the corresponding annotated translational start sites. In addition, 216 proteins were identified with unmodified N‐termini, of which 163 cases represented peptides with cleavage of the initiator methionine. In the case of the HNRNPA3 protein, we detected two N‐terminally acetylated peptides (Ac‐MEVKPPPGRPQPDSGR and Ac‐MEGHDPKEPEQLR). These peptides map to two known isoforms (NX_P51991‐1 and NX_P51991‐2) of the HNRNPA3 protein, which confirms the presence of alternate translational start sites for this protein. The number of PSMs for the acetylated N‐terminal peptide specific to the NX_P51991‐1 was twice as compared to that from NX_P51991‐2. However, the relative abundance of the corresponding mRNAs was three time for NX_P51991‐1 as compared to NX_P51991‐2. This observation also implicates the transcriptional and post‐transcriptional regulation involved in differential expression and translation of isoforms for gene(s).

**Figure 5:**
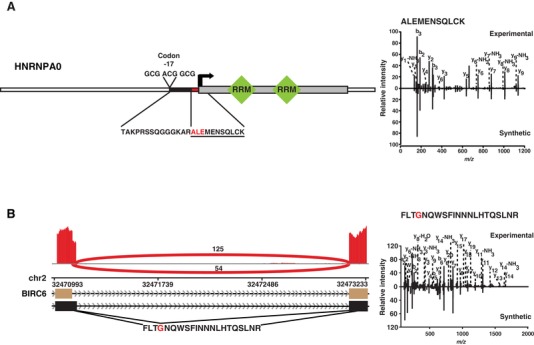
Schematic representation of alternative transcript expression. A) HNRNPA0 protein identified with an upstream alternate N‐terminus in‐frame with annotated start site (bent arrow) is shown. B) Alternative splice donor in *BIRC6* is supported by RNA‐seq and novel junctional peptide. The annotated MS/MS spectra supporting these finding is also shown. Known and novel transcript models are shown in brown and black colors, respectively. Track in red color shows the sashimi plot with thick curves connecting the exon–exon boundaries. Amino acid that span the splicing junction are marked in red.

We also identified peptides upstream of annotated translational start sites in the annotated 5’‐UTR regions of seven proteins FXR2, NPLOC4, MINOS1, SFPQ, RHOB, ATP9A, and HNRNPA0. FXR2 and NPLOC4 were identified with an upstream peptide, which was in‐frame with annotated start site (Figure S1, Supporting Information). Recent studies have also reported putative alternate N‐termini for these proteins.^67^, ^71^, ^72^ For the remaining five proteins, we identified peptides that were direct extensions from annotated protein N‐termini into the 5’‐UTR region which suggests the existence of an alternate N‐terminus or multiple N‐termini. We looked for the presence of canonical and non‐canonical start codons upstream of identified peptides in the 5’‐UTR and identified near cognate TIS in all five cases. Notably, in two out of five novel cases we found supporting evidence of alternate TIS in a previous Ribo‐seq study.^73^ A complete list of alternate N‐terminal extensions identified in this study is provided in Table 2. As an example, HNRNPA0 which was identified with a novel peptide (ALEMENSQLCK) extending from annotated protein N‐terminus and predicted to contain multiple alternate TIS at codons (‐51, ‐61, and ‐237) upstream of annotated TIS is shown in Figure 5A. Our results clearly demonstrate the existence of alternate N‐termini of proteins that can be specifically mined through the approach employed here.

**Table 2:** List of peptides identified upstream of annotated protein N‐termini

	Sequence	Gene symbol	Peptide position (from TIS)	Near cognate TIS position (codon)	Evidence from Ribo‐seq study
1.	VGNMSESELGR	*MINOS1*	−3	−7 (CTG)	
2.	FCLDRPLTTDMSR	*SFPQ*	−10	−21 (GUG)	73
3.	AAAADGERPGPGPLLVGCGR	*FXR2*	−68	−73 (GTG)	73
4.	EAGAGAEAAAGSARPLGR	*NPLOC4*	−34	−43 (CUG)	
5.	EGSEAFAGPLLLPGPGPLMAAIR	*RHOB*	−18	−23 (GUG)	
6.	AGGAADMTDNIPLQPVR	*ATP9A*	−6	−47 (CUG)	
7.	ALEMENSQLCK	*HNRNPA0*	−3	−17 (ACG)	73

TIS, translation initiation site.

Functional complexity of cellular systems is also determined by the alternate expression of transcript isoforms of annotated protein‐coding genes. Integrated analysis of mRNA and proteomics data allows us to study the differential regulation involved in splicing and translation of isoforms to derive novel proteoforms. To identify the cell‐type‐specific novel proteoforms, we carried out integrated analysis of transcriptomics and proteomics data. From a database search against the translated transcript assemblies and novel exon–exon junctions, we detected five proteins with peptides spanning the junctions from the proteomics data. For example, an alternate splice donor or acceptor splice junctions were identified for the proteins BIRC6 and PGAM5, respectively. Figure 5B shows the alternate splice donor in BIRC6 which extends the annotated exon by 24 bp into the intron with peptide sequence that spans the novel exon–exon junction and supporting MS/MS spectra. The proportion of reads that mapped to this novel exon–exon junction in BIRC6 were more than double those mapped against known exon–exon junctions suggesting possible differential regulation of isoforms. In the case of the *CCDC9* gene, we identified an alternate transcript that extends from the annotated last exon. The alternate transcript assembled from the RNA‐seq data clearly showed three additional exons located 3’ to the annotated last exon arising from a novel splice donor in the last annotated exon. The extended protein is also in‐frame with the protein encoded by the last annotated exon. The transcript model of the extended region from the annotated last exon as observed from the RNA‐seq reads mapped and the deduced amino acid sequence is shown in Figure S2A, Supporting Information. The identified peptide (underlined) spans the first novel exon–exon junction into the extended region. Notably, this novel transcript extension was also observed as a transcript in many mammals including monkey, mouse, dog, and elephant. We identified two proteins, PPP1R35 and CHMP2A, with canonical and an alternate isoform with intron retention at both transcript and protein levels. Figure S2B, Supporting Information shows a schematic of intron retention in CHMP2A. The reason why we did not identify an even greater number of novel proteoforms is likely because the abundance of these isoforms is relatively low in comparison to the major isoforms. This is in agreement with the previous studies which reported that 85% of total mRNA expression of genes was contributed by a single major isoform.^74^

## 4 Conclusions

The transcriptome and proteome undergo dynamic changes in cells that ultimately determine their structures and functions. While a large number of transcripts can be detected in cells/tissues by next‐generation sequencing, proteomic studies cannot identify those that might be specific to certain cells/tissues as database searches are carried out only against reference protein databases. Another caveat of standard proteomic analysis pipeline is that peptides with variant amino acids that result from coding SNPs cannot be identified if the databases being searched are not supplemented with a list of variants. Thus, the most optimal approach is to carryout RNA‐seq and proteomics analysis of same sample to maximize the advantages of a proteogenomics analysis. In this study, we carried out database searches against custom protein sequences that incorporated cSNPs and transcript variants from the RNA‐seq data. This allowed us to identify 245 additional peptides with sample‐specific sequence variants. Approaches that have been employed by most proteogenomics studies use common cSNP databases that contain variants that may not be present in the sample at all and thus could lead to false‐positive peptide identifications. Novel isoforms identified from transcriptomic analysis are also relevant for identification of novel proteoforms, which contain novel exon–exon junctions, some of which might be cell/tissue type specific and thus would not be identified in standard database searches. This approach is quite efficient in the case of a homogeneous cell type while tissues that comprise of many different cell types may require very deep sequencing.

Our approach also ensures that the focus remains on mRNA and protein species that are actually expressed in the cell type of interest. This strategy becomes even more relevant in the case of identifying missing proteins whose expression may be questionable. Even though integrated analysis is appealing and provides unique advantages, this approach has not been widely adopted because of the added cost of RNA‐seq, a good understanding of transcriptomics and proteomics, and the lack of data integration tools. In addition, visualization of integrated datasets for making interpretations is quite limited or simply unavailable for most proteomics researchers. We believe the results demonstrated in this study clearly demonstrate and emphasize the need to carry out integrated analysis of samples.

## Conflict of Interest

The authors declare no conflict of interest.

## Supporting information

Supplementary Figure S1. N‐terminal extension into 5’‐UTR regions of NPLOC4 (A) and FXR2 (B) proteins is shown along with their putative near‐cognate TIS and non‐canonical codons.

Supplementary Figure S2. (A) Alternative transcript of CCDC9 resulting from the extension of annotated last exon into three new downstream exons is shown. Bottom two tracks show sequence similarly across species of these novel exons (B) Intron retention in case of CHMP2A is shown along with the peptide. Known and novel transcript models are shown in brown and black color respectively. Track in red color shows the sashimi plot with thick curves connecting the exon‐exon boundaries. Amino acids that span the splicing junction are marked in red.

Supporting Information

Supporting Information

Supporting Information

Supporting Information

Supporting Information
